# Crystal structure and Hirshfeld surface analysis of 1-{[2-oxo-3-(prop-1-en-2-yl)-2,3-di­hydro-1*H*-1,3-benzo­diazol-1-yl]meth­yl}-3-(prop-1-en-2-yl)-2,3-di­hydro-1*H*-1,3-benzo­diazol-2-one

**DOI:** 10.1107/S2056989018015219

**Published:** 2018-11-09

**Authors:** Asmaa Saber, Nada Kheira Sebbar, Tuncer Hökelek, Brahim Hni, Joel T. Mague, El Mokhtar Essassi

**Affiliations:** a Laboratoire de Chimie Organique Hétérocyclique URAC 21, Pôle de Compétence Pharmacochimie, Av. Ibn Battouta, BP 1014, Faculté des Sciences, Université Mohammed V, Rabat, Morocco; b Laboratoire de Chimie Bioorganique Appliquée, Faculté des Sciences, Université Ibn Zohr, Agadir, Morocco; c Department of Physics, Hacettepe University, 06800 Beytepe, Ankara, Turkey; d Department of Chemistry, Tulane University, New Orleans, LA 70118, USA

**Keywords:** crystal structure, hydrogen bond, benzo­diazo­lone, Hirshfeld surface

## Abstract

In the title compound, C_21_H_20_N_4_O_2_, the intra­molecular C—H⋯O hydrogen-bonded benzo­diazo­lone moieties are planar to within 0.017 (1) and 0.026 (1) Å, and are oriented at a dihedral angle of 57.35 (3)°. In the crystal, two sets of inter­molecular C—H⋯O hydrogen bonds generate layers parallel to the *bc* plane. The Hirshfeld surface analysis of the crystal structure indicates that the most important contributions for the crystal packing are from H⋯H (51.8%), H⋯C/C⋯H (30.7%) and H⋯O/O⋯H (11.2%) inter­actions.

## Abstract 2

In the title compound, the benzo­diazo­lone moieties are planar to within 0.017 (1) and 0.026 (1) Å, and oriented at a dihedral angle of 57.35 (3)°. In the crystal, two sets of inter­molecular C—H⋯O hydrogen bonds generate layers parallel to the *bc* plane.

## Chemical context   

The benzimidazole unit is an important pharmacophore and a privileged structure in the functions of biological mol­ecules. Benzimidazole derivatives have attracted considerable attention from researchers because their bioactive and pharmaceutical properties. Many members of this family are widely used as anti­convulsant, anti-fungal, analgesic, anti­microbial, anti-histaminic and hypnotic or anti-inflammatory agents (Ayhan-Kılcıgil *et al.*, 2007 ▸; Soderlind *et al.*, 1999 ▸; Luo *et al.*, 2011 ▸; Walia *et al.*, 2011 ▸; Navarrete-Vázquez *et al.*, 2001 ▸). Benzimid­azolone derivatives also find commercial use as dyes for acrylic fibres. The search for new heterocyclic systems including the benzimidazolone moiety with biological activities therefore is of much current importance (Mondieig *et al.*, 2013 ▸; Lakhrissi *et al.*, 2008 ▸; Ouzidan *et al.*, 2011 ▸; Dardouri *et al.*, 2011 ▸). In this context, we are inter­ested in the synthesis of the title compound, 1-{[2-oxo-3-(prop-1-en-2-yl)2,3-di­hydro-1*H*-1,3-benzo­diazol-1-yl)meth­yl}-3-(prop-1-en-2-yl)-2,3-di­hydro-1*H*-1,3-benzo­diazol-2-one, by reaction of di­chloro­methane with 1-(prop-1-en-2-yl)-1*H*-benzimidazol-2(3*H*)-one under phase-transfer catalysis (PTC) conditions using tetra-*n*-butyl­ammonium bromide (TBAB) as catalyst and potassium carbonate as base. We report herein its crystal and mol­ecular structures along with the Hirshfeld surface analysis.

## Structural commentary   

**Figure 1:**
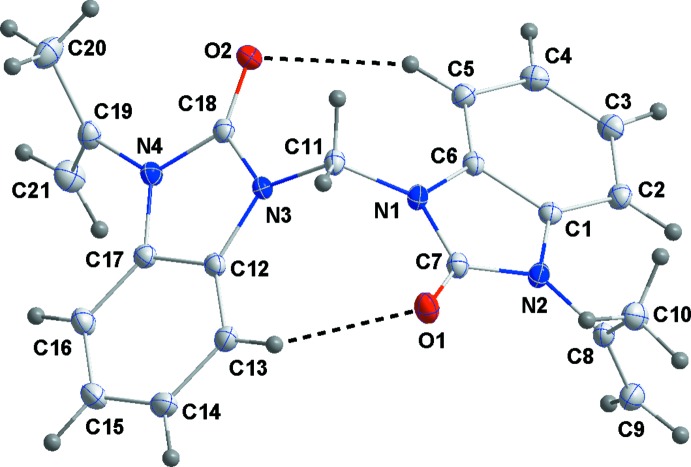
The title mol­ecule with the labelling scheme and 50% probability ellipsoids. Intra­molecular C—H⋯O hydrogen bonds are shown as dashed lines.

In the title compound (Fig. 1 ▸), the intra­molecular C—H⋯O hydrogen-bonded (Table 1 ▸) benzo­diazo­lone moieties are planar with the largest deviations being 0.017 (1) Å for atom C7 in the N1-containing unit (r.m.s. deviation = 0.011 Å) and 0.026 (1) Å for atom C18 in the N3-containing unit (r.m.s. deviation = 0.019 Å). The dihedral angle between the mean planes of the benzo­diazo­lone moieties is 57.35 (3)°.
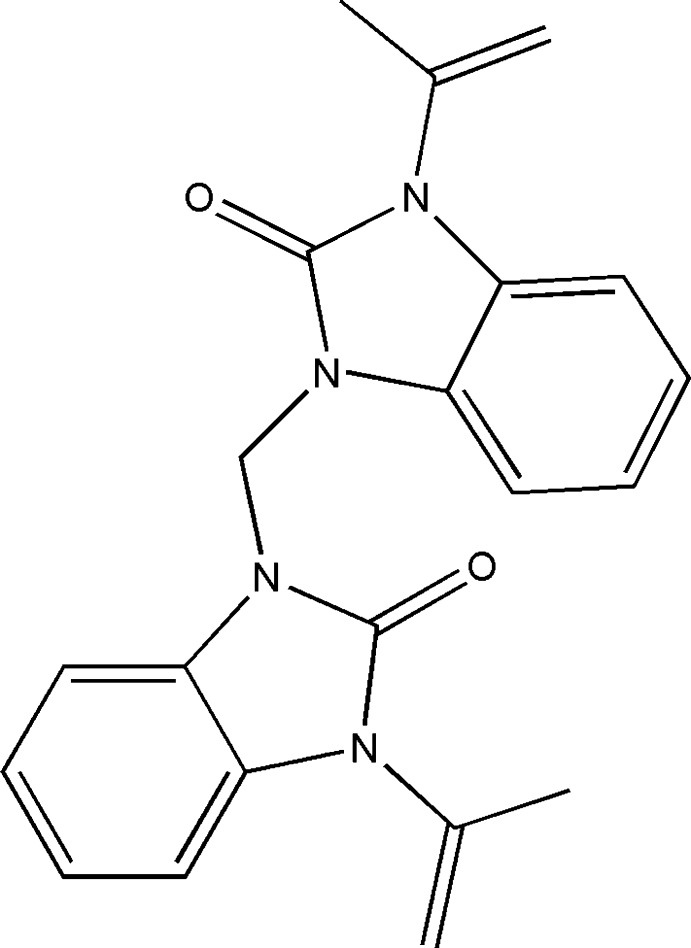


## Supra­molecular features   

**Figure 2:**
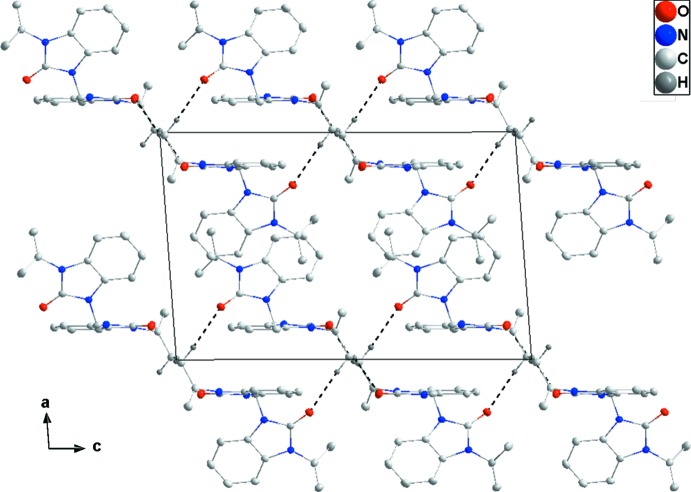
The packing viewed along the *b*-axis direction giving an elevation view of two adjacent layers. Inter­molecular C—H⋯O hydrogen bonds are shown as dashed lines.

Hydrogen bonding and van der Waals contacts are the dominant inter­actions in the crystal packing. In the crystal, two sets of inter­molecular C—H⋯O hydrogen bonds (Table 1 ▸) generate layers parallel to the *bc* plane. In these layers, one of the benzo­diazole units in each mol­ecule is approximately parallel to the *bc* plane while the other half of the mol­ecule protrudes from the surface (Fig. 2 ▸).

## Hirshfeld surface analysis   

**Figure 3:**
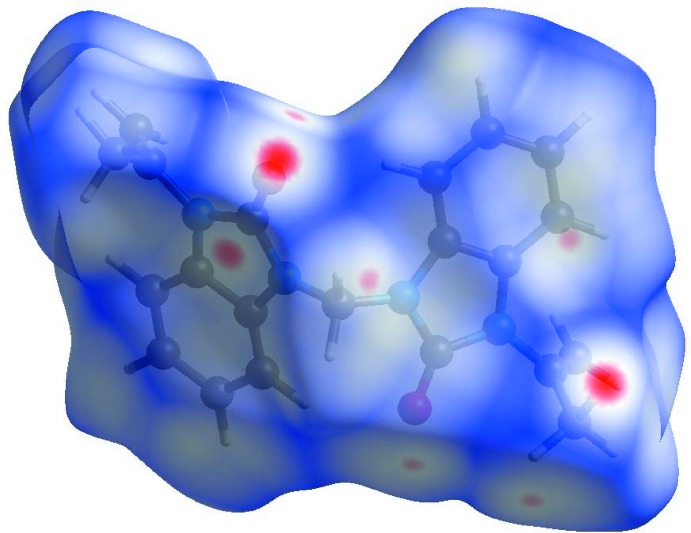
View of the three-dimensional Hirshfeld surface of the title compound plotted over *d*_norm_ in the range −0.1476 to 1.2686 a.u.

**Figure 4:**
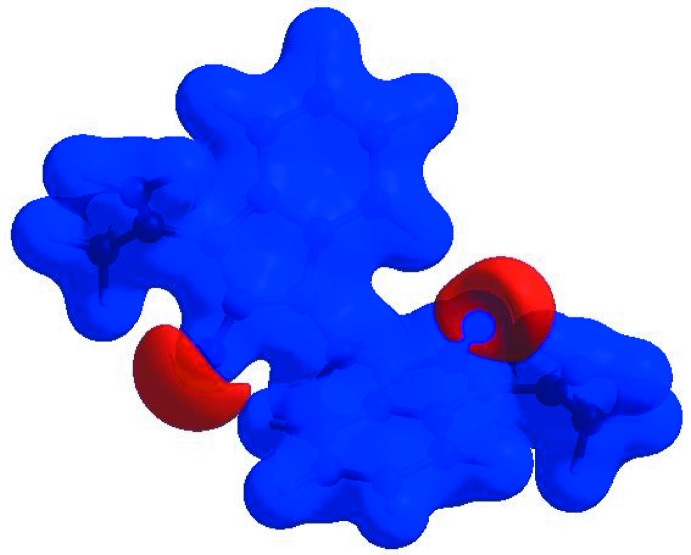
View of the three-dimensional Hirshfeld surface of the title compound plotted over electrostatic potential energy in the range −0.0500 to 0.0500 a.u. using the STO-3 G basis set at the Hartree–Fock level of theory. Hydrogen-bond donors and acceptors are shown as blue and red regions, respectively, around the atoms corresponding to positive and negative potentials.

**Figure 5:**
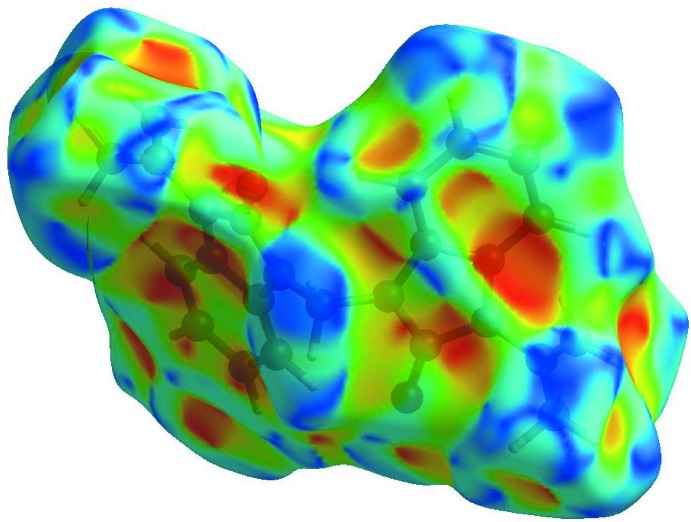
Hirshfeld surface of the title compound plotted over shape-index.

In order to visualize the inter­molecular inter­actions in the crystal of the title compound, a Hirshfeld surface (HS) analysis (Hirshfeld, 1977 ▸; Spackman & Jayatilaka, 2009 ▸) was carried out by using *CrystalExplorer17.5* (Turner *et al.*, 2017 ▸). In the HS plotted over *d*_norm_ (Fig. 3 ▸), the white surface indicates contacts with distances equal to the sum of van der Waals radii, and the red and blue colours indicate distances shorter (in close contact) or longer (distinct contact) than the van der Waals radii (Venkatesan *et al.*, 2016 ▸). The bright-red spots appearing near O1, O2 and hydrogen atoms H5, H10*A* and H10*B* indicate their roles as the respective donors and acceptors in the dominant C—H ⋯ O hydrogen bonds; they also appear as blue and red regions corresponding to positive and negative potentials on the HS mapped over electrostatic potential (Spackman *et al.*, 2008 ▸; Jayatilaka *et al.*, 2005 ▸) as shown in Fig. 4 ▸. The blue regions indicate positive electrostatic potential (hydrogen-bond donors), while the red regions indicate negative electrostatic potential (hydrogen-bond acceptors). The shape-index of the HS is a tool to visualize the π–π stacking by the presence of adjacent red and blue triangles; if there are no adjacent red and/or blue triangles, then there are no π–π inter­actions. Fig. 5 ▸ clearly indicates that no π–π inter­actions are present in the title structure.

**Figure 6:**
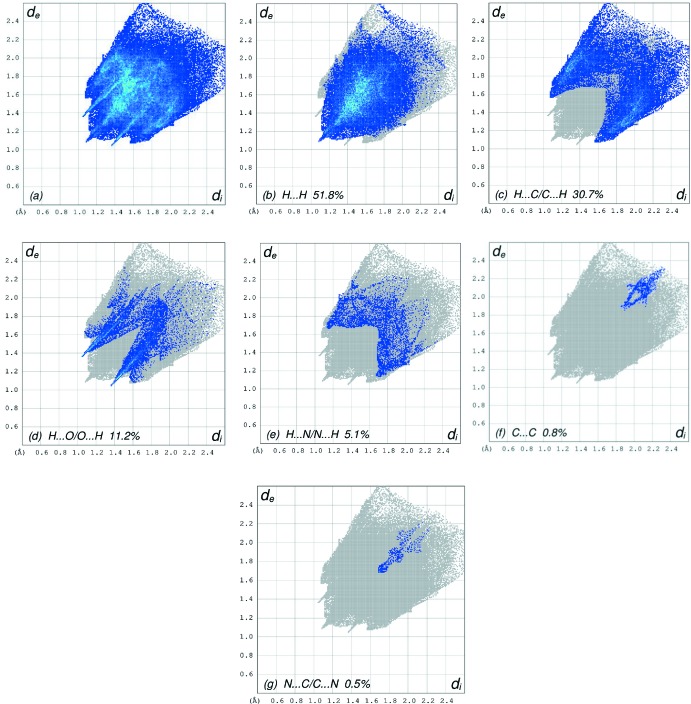
The full two-dimensional fingerprint plots for the title compound, showing (*a*) all inter­actions, and delineated into (*b*) H⋯H, (*c*) H⋯C/C⋯H, (*d*) H⋯O/O⋯H, (*e*) H⋯N/N⋯H, (*f*) C⋯C and (*g*) N⋯C/C⋯N inter­actions. The *d*_i_ and *d*_e_ values are the closest inter­nal and external distances (in Å) from given points on the Hirshfeld surface.

The overall two-dimensional fingerprint plot, Fig. 6 ▸ *a*, and those delineated into H⋯H, H⋯C/C⋯H, H⋯O/O⋯H, H⋯N/N⋯H, C⋯C and N⋯C/C⋯N contacts (McKinnon *et al.*, 2007 ▸) are illustrated in Fig. 6 ▸ *b*–*g*, respectively, together with their relative contributions to the Hirshfeld surface. The most important contribution to the overall crystal packing (51.8%) is from H⋯H inter­actions, which are shown in Fig. 6 ▸ *b* as widely scattered points of high density due to the large hydrogen content of the mol­ecule. The spike with the tip at *d*_e_ = *d*_i_ = 1.08 Å in Fig. 6 ▸ *b* is due to the short inter­atomic H⋯H contacts (Table 2 ▸). The fingerprint plot, Fig. 6 ▸ *c*, delineated into H⋯C/C⋯H contacts, which make a 30.7% contribution to the HS, shows a pair of characteristic wings and a pair of spikes with the tips at *d*_e_ + *d*_i_ ∼2.65 Å. The H⋯O/O⋯H contacts in the structure with a 11.2% contribution to the HS have a symmetrical distribution of points, Fig. 6 ▸ *d*, with the tips at *d*_e_ + *d*_i_ = 2.40 Å arising from the short intra- and/or inter­atomic C—H ⋯ O hydrogen bonding (Table 1 ▸) as well as from the H⋯O/O⋯H contacts (Table 2 ▸). Finally, the H⋯N/N⋯H (Fig. 6 ▸ *e*) contacts in the structure with a 5.1% contribution to the HS also have a symmetrical distribution of points, with the pair of wings appearing at *d*_e_ + *d*_i_ = 2.80 Å.

**Figure 7:**
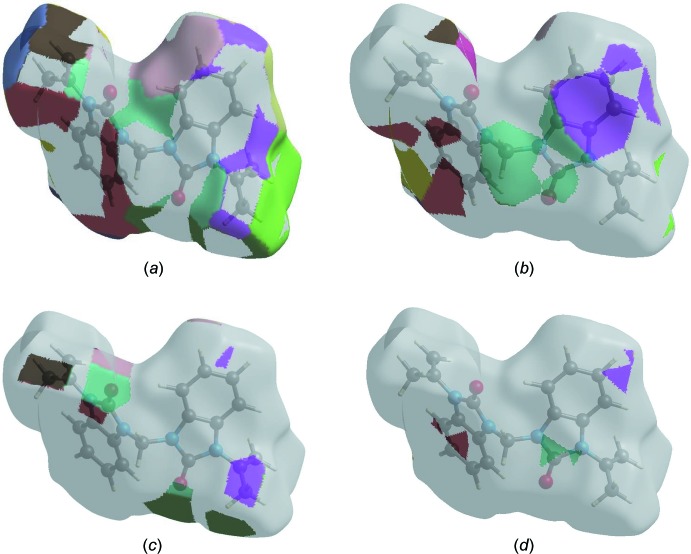
The Hirshfeld surface representations with the function *d*_norm_ plotted onto the surface for (*a*) H⋯H, (*b*) H⋯C/C⋯H, (*c*) H⋯O/O⋯H and (*d*) H⋯N/N⋯H inter­actions.

The Hirshfeld surface representations for the function d_norm_ are shown for the H⋯H, H⋯C/C⋯H, H⋯O/O⋯H and H⋯N/N⋯H inter­actions in Fig. 7 ▸ *a*–*d*, respectively.

The Hirshfeld surface analysis confirms the importance of H-atom contacts in establishing the packing. The large number of H⋯H, H⋯C/C⋯H and H⋯O/O⋯H inter­actions suggest that van der Waals inter­actions and hydrogen bonding play the major roles in the crystal packing (Hathwar *et al.*, 2015 ▸).

## Database survey   

**Figure 8:**
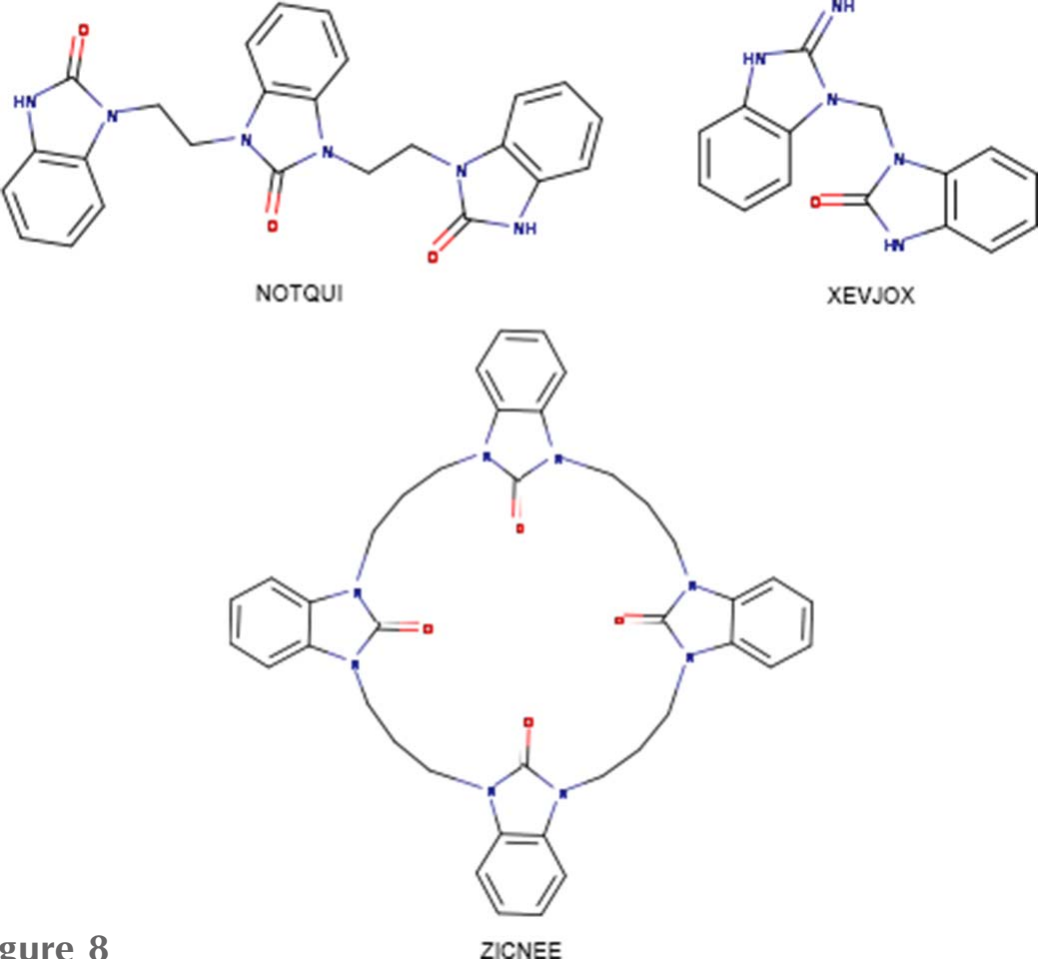
NOTQUI, XEVJOX and ZICNEE.

A search of the Cambridge Structural Database (CSD, version 5.39, update of August 2018; Groom *et al.*, 2016 ▸) for benzimidazolin-2-one derivatives in which both nitro­gen atoms form exocyclic C—N bonds gave 61 hits. In these structures, the bicyclic ring system is either planar, has a slight twist end-to-end or, in the cases where the exocyclic substituents form a ring, has a very shallow bowl shape. The closest examples to the title compound are NOTQUI (Díez-Barra *et al.*, 1997 ▸) and XEVJOX (Huang *et al.*, 2001 ▸) with ZICNEE (Shi & Thummel, 1995 ▸) as a more distant relative (see Fig. 8 ▸). In XEVJOX, the N—C—N angle connecting the two bicyclic units [114.19 (12)°] is essentially the same as in the title compound [114.04 (7)°]. In both of these, the bicyclic units are in an *anti* arrangement and this is basically the same for ZICNEE. Inter­estingly, the three bicyclic units in NOTQUI are close to all being *syn* to one another.

## Synthesis and crystallization   

To a solution of 1-(prop-1-en-2-yl)-1*H*-benzimidazol-2(3*H*)-one (2.87mmol) in di­chloro­methane (30 ml) as reagent and solvent were added potassium carbonate (5.71 mmol) and a catalytic amount of tetra-*n*-butyl­ammonium bromide (0.37 mmol). The mixture was heated for 24 h. The solid material was removed by filtration and the solvent evaporated under vacuum. The solid product was purified by recrystallization from ethanol solution to afford colourless crystals in 67% yield.

## Refinement   

Crystal data, data collection and structure refinement details are summarized in Table 3 ▸. H atoms were located in a difference-Fourier map and were freely refined.

## Supplementary Material

Crystal structure: contains datablock(s) I, global. DOI: 10.1107/S2056989018015219/xu5947sup1.cif

Structure factors: contains datablock(s) I. DOI: 10.1107/S2056989018015219/xu5947Isup2.hkl

Supporting information file. DOI: 10.1107/S2056989018015219/xu5947Isup3.cdx

Supporting information file. DOI: 10.1107/S2056989018015219/xu5947Isup4.cml

CCDC reference: 1875883

Additional supporting information:  crystallographic information; 3D view; checkCIF report

## Figures and Tables

**Table 1:** Hydrogen-bond geometry (Å, °)

*D*—H⋯*A*	*D*—H	H⋯*A*	*D*⋯*A*	*D*—H⋯*A*
C5—H5⋯O2	0.951 (14)	2.493 (14)	3.3598 (11)	151.4 (10)
C10—H10*A*⋯O1^i^	0.986 (14)	2.596 (14)	3.3498 (12)	133.3 (11)
C10—H10*B*⋯O2^vi^	0.998 (14)	2.488 (14)	3.4780 (12)	171.4 (11)
C13—H13⋯O1	0.961 (13)	2.477 (13)	3.3381 (11)	149.1 (11)

Symmetry codes: (i) 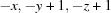; (vi) 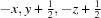.

**Table 2:** Selected interatomic distances (Å)

O1⋯C10	3.2233 (12)	C3⋯H11*A* ^vi^	3.017 (12)
O1⋯C13	3.3381 (12)	C4⋯H20*B* ^vii^	3.013 (14)
O1⋯C10^i^	3.3499 (12)	C5⋯H11*A*	2.980 (12)
O2⋯C20	3.1500 (13)	C7⋯H13	3.084 (13)
O2⋯C5	3.3598 (12)	C7⋯H21*B* ^v^	2.962 (14)
O1⋯H11*B*	2.510 (12)	C7⋯H10*A*	2.891 (14)
O1⋯H13	2.477 (13)	C8⋯H2	2.956 (13)
O1⋯H10*A* ^i^	2.595 (14)	C9⋯H2	2.981 (13)
O1⋯H20*C* ^ii^	2.917 (15)	C9⋯H15^ix^	2.898 (13)
O1⋯H10*A*	2.667 (14)	C10⋯H9*B* ^x^	3.051 (14)
O2⋯H3^iii^	2.772 (13)	C11⋯H2^iii^	3.077 (13)
O2⋯H5	2.493 (14)	C11⋯H13	3.009 (13)
O2⋯H11*A*	2.505 (12)	C11⋯H5	2.972 (13)
O2⋯H20*C*	2.581 (15)	C13⋯H11*B*	2.965 (12)
O2⋯H10*B* ^iv^	2.487 (14)	C13⋯H9*A* ^iii^	3.051 (14)
O2⋯H15^v^	2.781 (14)	C14⋯H9*A* ^iii^	2.989 (14)
N1⋯C3^iv^	3.3814 (12)	C16⋯H21*A*	3.080 (14)
N2⋯C21^v^	3.4318 (13)	C17⋯H21*A*	2.979 (14)
N3⋯H3^iii^	2.938 (13)	C18⋯H16^v^	2.858 (14)
N3⋯H16^v^	2.920 (14)	C18⋯H5	3.093 (14)
C1⋯C21^v^	3.5839 (13)	C18⋯H20*C*	2.852 (15)
C2⋯C11^vi^	3.5159 (12)	C18⋯H3^iii^	2.701 (13)
C2⋯C9	3.4314 (13)	C19⋯H16	2.977 (14)
C3⋯C18^vii^	3.3908 (13)	C21⋯H16	2.874 (14)
C3⋯C11^vi^	3.3994 (12)	H2⋯H11*B* ^vii^	2.428 (18)
C7⋯C21^v^	3.5253 (13)	H5⋯H11*A*	2.583 (18)
C16⋯C21	3.3315 (14)	H9*B*⋯H10*C*	2.495 (19)
C16⋯C18^viii^	3.4599 (13)	H9*B*⋯H10*B* ^x^	2.44 (2)
C1⋯H21*A* ^v^	2.941 (14)	H9*B*⋯H15^ix^	2.578 (19)
C1⋯H9*A*	3.064 (13)	H10*C*⋯H9*B*	2.495 (19)
C1⋯H11*A* ^vi^	2.946 (12)	H20*A*⋯H21*B*	2.45 (2)
C2⋯H11*A* ^vi^	2.786 (12)	H20*A*⋯H20*A* ^xi^	2.34 (2)

Symmetry codes: (i) 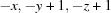; (ii) 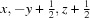; (iii) 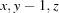; (iv) 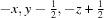; (v) 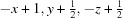; (vi) 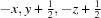; (vii) 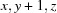; (viii) 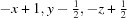; (ix) 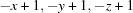; (x) 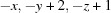; (xi) 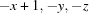.

**Table 3:** Experimental details

Crystal data	
Chemical formula	C_21_H_20_N_4_O_2_
*M*_r_	360.41
Crystal system, space group	Monoclinic, *P*2_1_/*c*
Temperature (K)	100
*a*, *b*, *c* (Å)	11.5244 (5), 8.6312 (4), 17.9845 (8)
β (°)	94.134 (1)
*V* (Å^3^)	1784.25 (14)
*Z*	4
Radiation type	Mo *K*α
μ (mm^−1^)	0.09
Crystal size (mm)	0.40 × 0.39 × 0.22

Data collection	
Diffractometer	Bruker SMART APEX CCD
Absorption correction	Multi-scan (*SADABS*; Krause *et al.*, 2015 ▸)
*T*_min_, *T*_max_	0.89, 0.98
No. of measured, independent and observed [*I* > 2σ(*I*)] reflections	33699, 4912, 4269
*R*_int_	0.027
(sin θ/λ)_max_ (Å^−1^)	0.696

Refinement	
*R*[*F* ^2^ > 2σ(*F* ^2^)], *wR*(*F* ^2^), *S*	0.039, 0.113, 1.09
No. of reflections	4912
No. of parameters	324
H-atom treatment	All H-atom parameters refined
Δρ_max_, Δρ_min_ (e Å^−3^)	0.47, −0.18

Computer programs: *APEX3* and *SAINT* (Bruker, 2016 ▸), *SHELXT* (Sheldrick, 2015*a* ▸), *SHELXL2018* (Sheldrick, 2015*b* ▸), *DIAMOND* (Brandenburg & Putz, 2012 ▸) and *SHELXTL* (Sheldrick, 2008 ▸).

## supplementary crystallographic information

### Crystal data

**Table:**

C_21_H_20_N_4_O_2_	*F*(000) = 760
*M**_r_* = 360.41	*D*_x_ = 1.342 Mg m^−^^3^
Monoclinic, *P*2_1_/*c*	Mo *K*α radiation, λ = 0.71073 Å
*a* = 11.5244 (5) Å	Cell parameters from 9896 reflections
*b* = 8.6312 (4) Å	θ = 2.3–29.6°
*c* = 17.9845 (8) Å	µ = 0.09 mm^−^^1^
β = 94.134 (1)°	*T* = 100 K
*V* = 1784.25 (14) Å^3^	Block, colourless
*Z* = 4	0.40 × 0.39 × 0.22 mm

### Data collection

**Table:**

Bruker SMART APEX CCD diffractometer	4912 independent reflections
Radiation source: fine-focus sealed tube	4269 reflections with *I* > 2σ(*I*)
Graphite monochromator	*R*_int_ = 0.027
Detector resolution: 8.3333 pixels mm^-1^	θ_max_ = 29.7°, θ_min_ = 1.8°
φ and ω scans	*h* = −15→15
Absorption correction: multi-scan (*SADABS*; Krause *et al.*, 2015)	*k* = −11→11
*T*_min_ = 0.89, *T*_max_ = 0.98	*l* = −25→25
33699 measured reflections	

### Refinement

**Table:**

Refinement on *F*^2^	Primary atom site location: structure-invariant direct methods
Least-squares matrix: full	Secondary atom site location: difference Fourier map
*R*[*F*^2^ > 2σ(*F*^2^)] = 0.039	Hydrogen site location: difference Fourier map
*wR*(*F*^2^) = 0.113	All H-atom parameters refined
*S* = 1.09	*w* = 1/[σ^2^(*F*_o_^2^) + (0.0731*P*)^2^ + 0.248*P*] where *P* = (*F*_o_^2^ + 2*F*_c_^2^)/3
4912 reflections	(Δ/σ)_max_ < 0.001
324 parameters	Δρ_max_ = 0.47 e Å^−^^3^
0 restraints	Δρ_min_ = −0.18 e Å^−^^3^

### Special details

**Table:**

Experimental. The diffraction data were obtained from 3 sets of 400 frames, each of width 0.5° in ω, colllected at φ = 0.00, 90.00 and 180.00° and 2 sets of 800 frames, each of width 0.45° in φ, collected at ω = –30.00 and 210.00°. The scan time was 15 sec/frame.
Geometry. All esds (except the esd in the dihedral angle between two l.s. planes) are estimated using the full covariance matrix. The cell esds are taken into account individually in the estimation of esds in distances, angles and torsion angles; correlations between esds in cell parameters are only used when they are defined by crystal symmetry. An approximate (isotropic) treatment of cell esds is used for estimating esds involving l.s. planes.
Refinement. Refinement of F^2^ against ALL reflections. The weighted R-factor wR and goodness of fit S are based on F^2^, conventional R-factors R are based on F, with F set to zero for negative F^2^. The threshold expression of F^2^ > 2sigma(F^2^) is used only for calculating R-factors(gt) etc. and is not relevant to the choice of reflections for refinement. R-factors based on F^2^ are statistically about twice as large as those based on F, and R- factors based on ALL data will be even larger.

### Fractional atomic coordinates and isotropic or equivalent isotropic displacement parameters (Å^2^)

**Table:**

	*x*	*y*	*z*	*U*_iso_*/*U*_eq_	
O1	0.14989 (6)	0.48310 (8)	0.43717 (4)	0.01960 (15)	
O2	0.23203 (6)	0.33229 (8)	0.13640 (4)	0.01728 (15)	
N1	0.15509 (6)	0.53341 (9)	0.30979 (4)	0.01421 (16)	
N2	0.14171 (6)	0.73261 (9)	0.38646 (4)	0.01478 (16)	
N3	0.26416 (6)	0.31586 (9)	0.26564 (4)	0.01347 (16)	
N4	0.39789 (6)	0.21797 (9)	0.19636 (4)	0.01465 (16)	
C1	0.14167 (7)	0.79263 (10)	0.31433 (5)	0.01364 (17)	
C2	0.13453 (8)	0.94353 (11)	0.28808 (5)	0.01628 (18)	
H2	0.1277 (11)	1.0298 (15)	0.3234 (7)	0.020 (3)*	
C3	0.13427 (8)	0.96453 (11)	0.21101 (5)	0.01748 (18)	
H3	0.1274 (10)	1.0686 (15)	0.1888 (7)	0.021 (3)*	
C4	0.14246 (8)	0.83917 (11)	0.16280 (5)	0.01769 (18)	
H4	0.1443 (11)	0.8573 (15)	0.1091 (7)	0.021 (3)*	
C5	0.15041 (8)	0.68681 (11)	0.18947 (5)	0.01611 (18)	
H5	0.1568 (11)	0.5978 (16)	0.1588 (7)	0.024 (3)*	
C6	0.14948 (7)	0.66652 (10)	0.26575 (5)	0.01342 (17)	
C7	0.14860 (7)	0.57243 (10)	0.38459 (5)	0.01462 (17)	
C8	0.12521 (8)	0.81967 (11)	0.45309 (5)	0.01582 (18)	
C9	0.20574 (8)	0.92203 (12)	0.47637 (5)	0.02068 (19)	
H9A	0.2757 (12)	0.9366 (16)	0.4506 (7)	0.027 (3)*	
H9B	0.1940 (12)	0.9911 (16)	0.5186 (7)	0.027 (3)*	
C10	0.01276 (8)	0.78748 (12)	0.48653 (5)	0.01963 (19)	
H10A	0.0075 (12)	0.6777 (17)	0.5012 (8)	0.029 (3)*	
H10B	−0.0541 (12)	0.8114 (16)	0.4499 (8)	0.026 (3)*	
H10C	0.0073 (11)	0.8515 (16)	0.5326 (8)	0.027 (3)*	
C11	0.15234 (7)	0.37476 (10)	0.28445 (5)	0.01449 (17)	
H11A	0.0994 (10)	0.3665 (14)	0.2396 (7)	0.016 (3)*	
H11B	0.1243 (11)	0.3110 (14)	0.3247 (7)	0.018 (3)*	
C12	0.35153 (7)	0.25387 (10)	0.31484 (5)	0.01369 (17)	
C13	0.36158 (8)	0.24451 (11)	0.39187 (5)	0.01675 (18)	
H13	0.3032 (11)	0.2870 (16)	0.4215 (7)	0.022 (3)*	
C14	0.46041 (9)	0.16942 (11)	0.42436 (5)	0.01995 (19)	
H14	0.4683 (12)	0.1616 (16)	0.4794 (8)	0.027 (3)*	
C15	0.54473 (8)	0.10818 (12)	0.38065 (5)	0.0208 (2)	
H15	0.6129 (12)	0.0591 (17)	0.4037 (7)	0.031 (3)*	
C16	0.53439 (8)	0.11942 (11)	0.30305 (5)	0.01861 (19)	
H16	0.5928 (11)	0.0792 (17)	0.2729 (8)	0.028 (3)*	
C17	0.43617 (7)	0.19291 (10)	0.27099 (5)	0.01441 (17)	
C18	0.29128 (7)	0.29272 (10)	0.19241 (5)	0.01344 (17)	
C19	0.45369 (8)	0.16277 (10)	0.13270 (5)	0.01655 (18)	
C20	0.38248 (9)	0.05439 (13)	0.08298 (6)	0.0238 (2)	
H20A	0.4249 (13)	0.0262 (18)	0.0389 (8)	0.040 (4)*	
H20B	0.3693 (12)	−0.0429 (18)	0.1112 (8)	0.034 (4)*	
H20C	0.3068 (13)	0.0959 (18)	0.0661 (8)	0.033 (3)*	
C21	0.56303 (9)	0.20343 (13)	0.12450 (6)	0.0232 (2)	
H21A	0.6039 (12)	0.2754 (17)	0.1589 (8)	0.028 (3)*	
H21B	0.6045 (12)	0.1602 (16)	0.0827 (8)	0.030 (3)*	

### Atomic displacement parameters (Å^2^)

**Table:**

	*U*^11^	*U*^22^	*U*^33^	*U*^12^	*U*^13^	*U*^23^
O1	0.0239 (3)	0.0166 (3)	0.0187 (3)	0.0040 (3)	0.0045 (3)	0.0038 (2)
O2	0.0170 (3)	0.0173 (3)	0.0171 (3)	0.0021 (2)	−0.0019 (2)	0.0004 (2)
N1	0.0163 (3)	0.0111 (4)	0.0153 (3)	0.0012 (3)	0.0022 (3)	−0.0005 (3)
N2	0.0180 (3)	0.0121 (4)	0.0143 (3)	0.0011 (3)	0.0021 (3)	−0.0008 (3)
N3	0.0124 (3)	0.0133 (3)	0.0146 (3)	0.0022 (3)	0.0004 (3)	−0.0010 (3)
N4	0.0134 (3)	0.0157 (4)	0.0149 (3)	0.0026 (3)	0.0016 (3)	−0.0004 (3)
C1	0.0114 (4)	0.0139 (4)	0.0156 (4)	−0.0004 (3)	0.0006 (3)	−0.0006 (3)
C2	0.0157 (4)	0.0132 (4)	0.0198 (4)	−0.0015 (3)	0.0004 (3)	−0.0014 (3)
C3	0.0171 (4)	0.0145 (4)	0.0206 (4)	−0.0030 (3)	−0.0002 (3)	0.0024 (3)
C4	0.0173 (4)	0.0187 (5)	0.0169 (4)	−0.0025 (3)	0.0001 (3)	0.0022 (3)
C5	0.0158 (4)	0.0163 (4)	0.0162 (4)	−0.0006 (3)	0.0008 (3)	−0.0016 (3)
C6	0.0111 (4)	0.0116 (4)	0.0175 (4)	0.0000 (3)	0.0007 (3)	−0.0003 (3)
C7	0.0129 (4)	0.0142 (4)	0.0169 (4)	0.0016 (3)	0.0024 (3)	−0.0005 (3)
C8	0.0178 (4)	0.0159 (4)	0.0138 (4)	0.0052 (3)	0.0008 (3)	−0.0013 (3)
C9	0.0204 (4)	0.0202 (5)	0.0213 (4)	0.0016 (4)	0.0007 (3)	−0.0049 (4)
C10	0.0173 (4)	0.0237 (5)	0.0181 (4)	0.0029 (3)	0.0031 (3)	−0.0017 (4)
C11	0.0114 (4)	0.0116 (4)	0.0206 (4)	0.0004 (3)	0.0018 (3)	−0.0020 (3)
C12	0.0134 (4)	0.0107 (4)	0.0168 (4)	0.0002 (3)	−0.0003 (3)	0.0000 (3)
C13	0.0190 (4)	0.0146 (4)	0.0167 (4)	0.0006 (3)	0.0011 (3)	−0.0017 (3)
C14	0.0237 (5)	0.0180 (4)	0.0175 (4)	0.0007 (3)	−0.0030 (3)	0.0004 (3)
C15	0.0192 (4)	0.0198 (5)	0.0226 (4)	0.0039 (3)	−0.0039 (3)	0.0015 (4)
C16	0.0153 (4)	0.0186 (4)	0.0218 (4)	0.0035 (3)	0.0010 (3)	0.0002 (3)
C17	0.0142 (4)	0.0129 (4)	0.0160 (4)	−0.0002 (3)	0.0003 (3)	−0.0003 (3)
C18	0.0133 (4)	0.0108 (4)	0.0162 (4)	−0.0008 (3)	0.0012 (3)	−0.0011 (3)
C19	0.0181 (4)	0.0157 (4)	0.0163 (4)	0.0027 (3)	0.0044 (3)	0.0008 (3)
C20	0.0235 (5)	0.0256 (5)	0.0231 (4)	−0.0021 (4)	0.0070 (4)	−0.0083 (4)
C21	0.0180 (4)	0.0292 (5)	0.0230 (5)	0.0011 (4)	0.0053 (4)	0.0011 (4)

### Geometric parameters (Å, º)

**Table:**

O1—C7	1.2194 (11)	C8—C10	1.4936 (13)
O2—C18	1.2241 (11)	C9—H9A	0.967 (13)
N1—C7	1.3938 (11)	C9—H9B	0.982 (14)
N1—C6	1.3943 (11)	C10—H10A	0.986 (14)
N1—C11	1.4428 (11)	C10—H10B	0.998 (14)
N2—C7	1.3853 (12)	C10—H10C	1.001 (14)
N2—C1	1.3969 (11)	C11—H11A	0.978 (12)
N2—C8	1.4386 (11)	C11—H11B	0.982 (12)
N3—C18	1.3899 (11)	C12—C13	1.3843 (12)
N3—C12	1.3980 (11)	C12—C17	1.4003 (12)
N3—C11	1.4478 (11)	C13—C14	1.4010 (13)
N4—C18	1.3850 (11)	C13—H13	0.961 (13)
N4—C17	1.3991 (11)	C14—C15	1.3970 (14)
N4—C19	1.4343 (11)	C14—H14	0.991 (14)
C1—C2	1.3858 (12)	C15—C16	1.3956 (13)
C1—C6	1.4026 (12)	C15—H15	0.960 (14)
C2—C3	1.3976 (13)	C16—C17	1.3863 (12)
C2—H2	0.986 (12)	C16—H16	0.959 (14)
C3—C4	1.3940 (13)	C19—C21	1.3265 (13)
C3—H3	0.984 (13)	C19—C20	1.4975 (14)
C4—C5	1.4005 (13)	C20—H20A	0.992 (15)
C4—H4	0.980 (13)	C20—H20B	0.998 (15)
C5—C6	1.3839 (12)	C20—H20C	0.971 (15)
C5—H5	0.951 (14)	C21—H21A	0.973 (14)
C8—C9	1.3271 (14)	C21—H21B	0.993 (15)
			
O1···C10	3.2233 (12)	C3···H11A^vi^	3.017 (12)
O1···C13	3.3381 (12)	C4···H20B^vii^	3.013 (14)
O1···C10^i^	3.3499 (12)	C5···H11A	2.980 (12)
O2···C20	3.1500 (13)	C7···H13	3.084 (13)
O2···C5	3.3598 (12)	C7···H21B^v^	2.962 (14)
O1···H11B	2.510 (12)	C7···H10A	2.891 (14)
O1···H13	2.477 (13)	C8···H2	2.956 (13)
O1···H10A^i^	2.595 (14)	C9···H2	2.981 (13)
O1···H20C^ii^	2.917 (15)	C9···H15^ix^	2.898 (13)
O1···H10A	2.667 (14)	C10···H9B^x^	3.051 (14)
O2···H3^iii^	2.772 (13)	C11···H2^iii^	3.077 (13)
O2···H5	2.493 (14)	C11···H13	3.009 (13)
O2···H11A	2.505 (12)	C11···H5	2.972 (13)
O2···H20C	2.581 (15)	C13···H11B	2.965 (12)
O2···H10B^iv^	2.487 (14)	C13···H9A^iii^	3.051 (14)
O2···H15^v^	2.781 (14)	C14···H9A^iii^	2.989 (14)
N1···C3^iv^	3.3814 (12)	C16···H21A	3.080 (14)
N2···C21^v^	3.4318 (13)	C17···H21A	2.979 (14)
N3···H3^iii^	2.938 (13)	C18···H16^v^	2.858 (14)
N3···H16^v^	2.920 (14)	C18···H5	3.093 (14)
C1···C21^v^	3.5839 (13)	C18···H20C	2.852 (15)
C2···C11^vi^	3.5159 (12)	C18···H3^iii^	2.701 (13)
C2···C9	3.4314 (13)	C19···H16	2.977 (14)
C3···C18^vii^	3.3908 (13)	C21···H16	2.874 (14)
C3···C11^vi^	3.3994 (12)	H2···H11B^vii^	2.428 (18)
C7···C21^v^	3.5253 (13)	H5···H11A	2.583 (18)
C16···C21	3.3315 (14)	H9B···H10C	2.495 (19)
C16···C18^viii^	3.4599 (13)	H9B···H10B^x^	2.44 (2)
C1···H21A^v^	2.941 (14)	H9B···H15^ix^	2.578 (19)
C1···H9A	3.064 (13)	H10C···H9B	2.495 (19)
C1···H11A^vi^	2.946 (12)	H20A···H21B	2.45 (2)
C2···H11A^vi^	2.786 (12)	H20A···H20A^xi^	2.34 (2)
			
C7—N1—C6	110.21 (7)	C8—C10—H10C	109.8 (8)
C7—N1—C11	122.19 (7)	H10A—C10—H10C	107.5 (11)
C6—N1—C11	127.12 (7)	H10B—C10—H10C	109.9 (11)
C7—N2—C1	110.10 (7)	N1—C11—N3	114.04 (7)
C7—N2—C8	123.56 (7)	N1—C11—H11A	109.1 (7)
C1—N2—C8	126.06 (8)	N3—C11—H11A	107.2 (7)
C18—N3—C12	110.11 (7)	N1—C11—H11B	107.4 (7)
C18—N3—C11	122.45 (7)	N3—C11—H11B	108.7 (7)
C12—N3—C11	126.81 (7)	H11A—C11—H11B	110.4 (10)
C18—N4—C17	109.78 (7)	C13—C12—N3	131.31 (8)
C18—N4—C19	124.12 (7)	C13—C12—C17	122.02 (8)
C17—N4—C19	125.86 (7)	N3—C12—C17	106.66 (7)
C2—C1—N2	131.38 (8)	C12—C13—C14	116.83 (8)
C2—C1—C6	121.47 (8)	C12—C13—H13	121.4 (8)
N2—C1—C6	107.15 (8)	C14—C13—H13	121.7 (8)
C1—C2—C3	117.10 (8)	C15—C14—C13	121.19 (9)
C1—C2—H2	119.7 (7)	C15—C14—H14	121.3 (8)
C3—C2—H2	123.2 (7)	C13—C14—H14	117.5 (8)
C4—C3—C2	121.38 (8)	C16—C15—C14	121.58 (9)
C4—C3—H3	117.6 (7)	C16—C15—H15	118.0 (8)
C2—C3—H3	121.0 (7)	C14—C15—H15	120.4 (8)
C3—C4—C5	121.44 (8)	C17—C16—C15	117.13 (8)
C3—C4—H4	119.7 (8)	C17—C16—H16	121.1 (8)
C5—C4—H4	118.8 (8)	C15—C16—H16	121.8 (8)
C6—C5—C4	116.95 (8)	C16—C17—N4	131.39 (8)
C6—C5—H5	118.6 (8)	C16—C17—C12	121.25 (8)
C4—C5—H5	124.4 (8)	N4—C17—C12	107.33 (7)
C5—C6—N1	131.61 (8)	O2—C18—N4	127.76 (8)
C5—C6—C1	121.65 (8)	O2—C18—N3	126.13 (8)
N1—C6—C1	106.75 (7)	N4—C18—N3	106.11 (7)
O1—C7—N2	127.56 (8)	C21—C19—N4	119.01 (9)
O1—C7—N1	126.66 (8)	C21—C19—C20	125.66 (9)
N2—C7—N1	105.77 (7)	N4—C19—C20	115.23 (8)
C9—C8—N2	118.63 (8)	C19—C20—H20A	110.5 (9)
C9—C8—C10	127.17 (8)	C19—C20—H20B	108.6 (8)
N2—C8—C10	114.13 (8)	H20A—C20—H20B	107.6 (12)
C8—C9—H9A	121.6 (8)	C19—C20—H20C	113.4 (9)
C8—C9—H9B	121.1 (8)	H20A—C20—H20C	108.9 (12)
H9A—C9—H9B	117.2 (12)	H20B—C20—H20C	107.6 (12)
C8—C10—H10A	110.9 (8)	C19—C21—H21A	121.2 (8)
C8—C10—H10B	110.3 (8)	C19—C21—H21B	119.9 (8)
H10A—C10—H10B	108.4 (11)	H21A—C21—H21B	118.9 (12)
			
C7—N2—C1—C2	178.97 (9)	C18—N3—C11—N1	−106.27 (9)
C8—N2—C1—C2	4.89 (14)	C12—N3—C11—N1	83.78 (10)
C7—N2—C1—C6	−0.36 (9)	C18—N3—C12—C13	−177.76 (9)
C8—N2—C1—C6	−174.44 (8)	C11—N3—C12—C13	−6.78 (15)
N2—C1—C2—C3	−178.63 (8)	C18—N3—C12—C17	1.01 (9)
C6—C1—C2—C3	0.62 (13)	C11—N3—C12—C17	171.99 (8)
C1—C2—C3—C4	−0.86 (13)	N3—C12—C13—C14	178.01 (9)
C2—C3—C4—C5	0.49 (14)	C17—C12—C13—C14	−0.60 (13)
C3—C4—C5—C6	0.15 (13)	C12—C13—C14—C15	0.41 (14)
C4—C5—C6—N1	179.51 (9)	C13—C14—C15—C16	0.17 (15)
C4—C5—C6—C1	−0.39 (13)	C14—C15—C16—C17	−0.55 (15)
C7—N1—C6—C5	−178.70 (9)	C15—C16—C17—N4	−177.31 (9)
C11—N1—C6—C5	−6.53 (15)	C15—C16—C17—C12	0.36 (14)
C7—N1—C6—C1	1.21 (9)	C18—N4—C17—C16	177.76 (9)
C11—N1—C6—C1	173.38 (8)	C19—N4—C17—C16	3.22 (16)
C2—C1—C6—C5	0.00 (13)	C18—N4—C17—C12	−0.16 (10)
N2—C1—C6—C5	179.41 (8)	C19—N4—C17—C12	−174.69 (8)
C2—C1—C6—N1	−179.92 (8)	C13—C12—C17—C16	0.22 (14)
N2—C1—C6—N1	−0.51 (9)	N3—C12—C17—C16	−178.69 (8)
C1—N2—C7—O1	−179.53 (8)	C13—C12—C17—N4	178.40 (8)
C8—N2—C7—O1	−5.27 (14)	N3—C12—C17—N4	−0.51 (9)
C1—N2—C7—N1	1.08 (9)	C17—N4—C18—O2	−179.70 (9)
C8—N2—C7—N1	175.34 (7)	C19—N4—C18—O2	−5.05 (15)
C6—N1—C7—O1	179.19 (8)	C17—N4—C18—N3	0.76 (10)
C11—N1—C7—O1	6.57 (13)	C19—N4—C18—N3	175.42 (8)
C6—N1—C7—N2	−1.42 (9)	C12—N3—C18—O2	179.36 (9)
C11—N1—C7—N2	−174.04 (7)	C11—N3—C18—O2	7.92 (14)
C7—N2—C8—C9	120.14 (10)	C12—N3—C18—N4	−1.10 (9)
C1—N2—C8—C9	−66.54 (12)	C11—N3—C18—N4	−172.54 (7)
C7—N2—C8—C10	−62.69 (11)	C18—N4—C19—C21	127.96 (10)
C1—N2—C8—C10	110.64 (10)	C17—N4—C19—C21	−58.25 (13)
C7—N1—C11—N3	−105.28 (9)	C18—N4—C19—C20	−55.46 (12)
C6—N1—C11—N3	83.41 (10)	C17—N4—C19—C20	118.33 (10)

Symmetry codes: (i) −*x*, −*y*+1, −*z*+1; (ii) *x*, −*y*+1/2, *z*+1/2; (iii) *x*, *y*−1, *z*; (iv) −*x*, *y*−1/2, −*z*+1/2; (v) −*x*+1, *y*+1/2, −*z*+1/2; (vi) −*x*, *y*+1/2, −*z*+1/2; (vii) *x*, *y*+1, *z*; (viii) −*x*+1, *y*−1/2, −*z*+1/2; (ix) −*x*+1, −*y*+1, −*z*+1; (x) −*x*, −*y*+2, −*z*+1; (xi) −*x*+1, −*y*, −*z*.

### Hydrogen-bond geometry (Å, º)

**Table:**

*D*—H···*A*	*D*—H	H···*A*	*D*···*A*	*D*—H···*A*
C5—H5···O2	0.951 (14)	2.493 (14)	3.3598 (11)	151.4 (10)
C10—H10*A*···O1^i^	0.986 (14)	2.596 (14)	3.3498 (12)	133.3 (11)
C10—H10*B*···O2^vi^	0.998 (14)	2.488 (14)	3.4780 (12)	171.4 (11)
C13—H13···O1	0.961 (13)	2.477 (13)	3.3381 (11)	149.1 (11)

Symmetry codes: (i) −*x*, −*y*+1, −*z*+1; (vi) −*x*, *y*+1/2, −*z*+1/2.
